# Association of High-Density Lipoprotein Cholesterol with the Estimated Glomerular Filtration Rate in a Community-Based Population

**DOI:** 10.1371/journal.pone.0079738

**Published:** 2013-11-06

**Authors:** Fan Wang, Jin Zheng, Ping Ye, Leiming Luo, Yongyi Bai, Ruyi Xu, Li Sheng, Tiehui Xiao, Hongmei Wu

**Affiliations:** Department of Geriatric Cardiology, Chinese PLA General Hospital, Beijing, China; University of São Paulo School of Medicine, Brazil

## Abstract

**Background:**

Reduced kidney function is independently associated with low high-density lipoprotein cholesterol (HDL-C) levels in patients with end-stage renal disease (ESRD), those on hemodialysis, and those with stage 3–5 chronic kidney disease (CKD). However, epidemiological data investigating the relationship between HDL-C levels and kidney function in the general population with roughly normal kidney function are limited, and the results are also inconsistent. The aim of this study was to evaluate the relationship between HDL-C levels and the estimated glomerular filtration rate (eGFR) in a community-based population in China.

**Methods:**

This was a community-based cross-sectional survey. In total, 4925 participants (age range, 18–96 years; mean, 51.30±11.98 years) were recruited during routine health status examinations. A questionnaire was used to ascertain age, smoking status, and the history of hypertension and diabetes mellitus for each participant. We measured the body mass index, waist circumference, systolic and diastolic blood pressure, and fasting glucose, total cholesterol, triglyceride, HDL-C, low-density lipoprotein cholesterol, uric acid, and serum creatinine level of each participant. eGFR was evaluated using the Chinese modified Modification of Diet in Renal Disease equation.

**Results:**

The HDL-C level was higher in the first quartile (lowest quartile) of eGFR than in the fourth quartile (the highest quartile). Additionally, HDL-C levels decreased as eGFR decreased. Pearson’s correlation analysis revealed that HDL-C levels were associated with eGFR (r=0.16). After adjustment for some confounders, HDL-C was independently associated with all quartiles of eGFR in the participants.

**Conclusions:**

HDL-C was independently associated with kidney function in a community-dwelling general population. The association between low HDL-C levels and a decreased eGFR gradually strengthened as eGFR declined.

## Introduction

Reduced kidney function is an independent risk factor for cardiovascular disease (CVD) and cardiac events [1]. Meanwhile, CVD risk factors such as age, hypertension, hyperglycemia, and dyslipidemia have effects on kidney function [2]. Previous studies demonstrated that reduced kidney function is independently associated with low high-density lipoprotein cholesterol (HDL-C) levels in patients with end-stage renal disease (ESRD), those on hemodialysis, and those with stage 3–5 chronic kidney disease (CKD) [3-5], and reduced HDL-C levels are a hallmark of ESRD-related dyslipidemia [6]. Some researchers are convinced that this correlation between kidney function and HDL-C is due to the dysregulation of HDL-C metabolism caused by reduced kidney function [7]. 

As an important cardiovascular risk factor, the cardioprotective effects of HDL-C have been confirmed by clinical and experimental research. However, it is unclear whether HDL-C has independent protective effects on kidney function. Epidemiological data investigating the relationship between HDL-C and kidney function in the general population with roughly normal kidney function are limited, and the results are also inconsistent [8,9]. Recently, Odden et al. reported that HDL-C was associated with a reduced cystatin C-based estimated glomerular filtration rate (eGFR) across the age spectrum, and the associations of HDL-C with reduced kidney function appeared to be stronger in older adults [10]. However, prior research indicated that after adjustment for age, HDL-C was not independently associated with kidney function. Therefore, in the present study, we investigated the relationship between HDL-C and creatinine-based eGFR in a community-based Chinese population. 

## Methods

The study protocol was approved by the Ethics Committee of the Chinese People’s Liberation Army General Hospital (Beijing, China). Each participant provided written informed consent to be included in the study.

### Study population

This community-based cross-sectional survey was designed to establish the relationship between HDL-C and eGFR through routine health status examinations conducted in three communities in Beijing (an urban community of the Shijingshan district, a town community, and a rural community of the Daxing district). The study population has been described previously [11]. The communities were selected by convenience, representing distinct economic, civilizational and lifestyle profiles (village, town and city). A minimum of 90% of residents in each community entered into the study and the participants were ethnically homogeneous (100% Han people). All participants were permanent residents, aged ≥18 years and were able to provide informed consent. Subjects with malignant tumours, bedridden status, mental disorders, severe heart and lung function failure or who were on dialysis were excluded from the study. Data collection was carried out between September 2007 and October 2008. A total of 5100 individuals (2111 from the urban community, 1513 from the rural community and 1476 from the town community) were eligible for inclusion in this survey. In the current paper, we excluded subjects with missing data for essential variables (n=175). Of these, 69 had missing height and weight measurements, 27 had missing systolic blood pressure (SBP) and diastolic blood pressure (DBP) measurements, 135 had missing serum creatinine (Scr) values, 120 had missing blood glucose (Glu), total cholesterol (TC), triglyceride (TG), low-density lipoprotein cholesterol (LDL-C) or HDL-C values, resulting in a final sample size of 4925.

### Questionnaire and anthropometric measurements

Information about the age, smoking status, the history of hypertension and diabetes mellitus, and medication use of the participants was obtained using standardized self-report questionnaires. This questionnaire was administered using a face-to-face counseling method. The investigation was completed by physicians in the Department of Geriatric Cardiology of the People’s Liberation Army General Hospital, who were trained by the research team. 

The physical examination included anthropometric and blood pressure measurements. Height, weight, and the waist and hip circumferences were measured. The body mass index (BMI) and waist-to-hip ratio (WHR) were calculated. BMI was calculated as the weight in kilograms divided by the height in meters squared (kg/m^2^). WHR was calculated as waist circumference (WC) divided by hip circumference. The blood pressure of the participants was measured in the sitting position. The blood pressure measurement was performed using a calibrated desktop sphygmomanometer (Yuyue, Armamentarium Limited Company, Jiangsu, China) after the participants had been in the sitting position for ≥5 min, consistent with current recommendations [12]. Blood pressure was measured three times consecutively, with ≥1 min between measurements. The mean value of blood pressure was used for the statistical analysis. 

### Laboratory measurements

Samples of venous blood were collected by venipuncture after an overnight fast. Blood samples (10 mL) were routinely stored at 4°C and delivered to the Department of Biochemistry and the laboratory of the Nephrology Department, People’s Liberation Army General Hospital on the same day. Concentrations of fasting glucose, total cholesterol, triglyceride, HDL-C, LDL-C, and uric acid were determined using the respective enzymatic assays (Roche Diagnostics GmbH, Mannheim, Germany) on an autoanalyzer (Roche Diagnostics, Indianapolis, IN, USA) in the Department of Biochemistry. Serum creatinine concentrations were measured using an enzymatic assay (Roche Diagnostics GmbH) on a Hitachi 7600 Autoanalyser (Hitachi, Tokyo, Japan) in the laboratory of the Nephrology Department. All testing was undertaken by well-trained personnel who were blinded to the clinical data.

### Definition of variables

Cigarette smoking was defined as smoking one cigarette per day and for a duration of at least 1 year. Hypertension was indicated by the following: (i) systolic blood pressure (SBP) ≥140 mmHg; (ii) diastolic blood pressure (DBP) ≥90 mmHg; and/or (iii) the use of an antihypertensive drug [13]. All participants without a history of diabetes mellitus (4559 participants) were given a standard 75 g oral glucose tolerance test (OGTT). Fasting venous blood was collected from participants with a history of diabetes mellitus to measure blood glucose. Diabetes mellitus was indicated by (i) a fasting glucose level ≥7.1 mmol/L, (ii) a 2 h venous blood glucose level ≥11.1 mmol/L, or (iii) the use of a hypoglycemic drug or insulin [14].

The following calibration equation was generated from the results (*R* ^2^=0.999) [15]: Jaffe’s kinetic method Scr (mg/dL)=0.795×[enzymatic method Scr (mg/dL)]+0.29. 

eGFR was calculated using the Chinese modified Modification of Diet in Renal Disease (CMDRD) equation as follows [16]: eGFR (mL/min/1.73 m^2^)=175×standard creatinine (mg/dL)^−1234^.×age (year)^−0.179^×0.79 (if female).

### Statistical analysis

Continuous variables are expressed as the mean and standard deviation (SD). Normality was tested using the Kolmogorov–Smirnov criterion. Skewed variables are expressed as the median value (with an interquartile range). Categorical variables are expressed as numbers and percentages. Baseline characteristics were separated according to the eGFR quartiles. eGFR levels were classified as follows: quartile 1 (≥102.35 mL/min/1.73 m^2^), quartile 2 (92.42–102.34 mL/min/1.73 m^2^), quartile 3 (83.67–92.41 ml/min/1.73 m^2^), and quartile 4 (≤83.66 mL/min/1.73 m^2^). Statistical comparison of the groups was undertaken by one-way ANOVA (continuous variables).

To evaluate the correlation between HDL-C levels and eGFR as a continuous variable, Pearson’s correlation analysis for continuous variables or Spearman’s correlation analysis for categorical variables was used in univariate analyses, and multivariable linear regression analysis was performed for variables with a probability value ≤0.10, and variables with a probability value <0.05 remained in the model after adjusting for several confounders (covariates). 

In addition, to better understand the correlation between HDL-C levels and different eGFR quartiles, logistic regression models were used. Forward stepwise multivariate logistic regression was performed to obtain the odds ratios (ORs) and 95% confidence intervals (CIs) for variables with a probability value ≤0.10, and those with a probability value <0.05 remained in the model after adjustment. Quartile 1 of eGFR was used as the reference. Regression models were adjusted for age and gender (model 1), and model 2 was adjusted for model 1 variables plus smoking status, a history of hypertension, and a history of diabetes mellitus. Model 3 was adjusted for model 2 variables plus BMI, WC, WHR, SBP, and DBP. Model 4 was adjusted for model 3 variables plus fasting glucose, triglyceride, LDL-C, and uric acid levels. 

All data entry and management activities were undertaken on an Excel spreadsheet, and data were analyzed using SAS statistical software (SAS Institute Incorporated, Cary, NC, USA), version 9.1. A 2-sided P-value <0.05 was considered significant. 

## Results

### Characteristics of participants

In total, 4925 subjects were included in the current analysis. The sample consisted of 2383 males (48.39%) and 2542 females (51.61%). The age range was 18–96 years, and the mean age was 51.30±11.98 years. There were 1483 smokers (30.11%), 571 participants with diabetes mellitus (11.59%) and 1751 hypertensive subjects (35.55%). Among the 4559 participants who completed the OGTT, 145 subjects were newly diagnosed with diabetes (2.94%). Three hundred seventeen subjects were newly diagnosed with hypertension (6.44%). 

Table 1 shows the clinical characteristics of the study participants. The participants were divided into four groups based on eGFR quartiles. Compared to quartile 1 of eGFR, participants in quartile 4 were more likely to be hypertensive and older, and they had higher values for SBP, total cholesterol, triglyceride, LDL-C, and uric acid (P<0.05) and lower values for HDL-C (P<0.05) Conversely, the percentage of males, smoking status, and the values for DBP and fasting glucose were not significantly different (P>0.05) between quartiles 1 and 4. Furthermore, HDL-C levels declined with decreasing eGFR values.

**Table 1:** The clinical characteristics of the study participants.

	**Overall**	**Quartile 1**	**Quartile 2**	**Quartile 3**	**Quartile 4**
	**(n=4925)**	**≥102.35**	**92.42–102.34**	**83.67–92.41**	**≤83.66**
**Characteristic**		**(n=1232)**	**(n=1230)**	**(n=1232)**	**(n=1231)**
eGFR	92.42	110.73	97.12	88.21	77.08
(ml/min/1.73 m^2^)	(83.66–102.36)	(106.30–117.69)	(94.76–99.62)	(86.05–90.22)	(71.00–80.60)
Age (years)	51.30±11.98	44.61±14.28	46.97±13.23*	52.55±13.32*	61.13±13.42**
male sex [n (%)]	2383 (48.3%)	584 (47.4%)	606 (49.3%)	602 (48.9%)	591 (48.0%)
Current smoking [n (%)]	1483 (30.1%)	368 (29.9%)	380 (30.9%)	350 (28.4%)	385 (31.3%)
Hypertension [n (%)]	1751 (35.6%)	360 (29.2%)	406 (33.0%)*	451 (36.6%)*	534 (43.4%)**
Diabetes mellitus [n (%)]	571 (11.6%)	121 (9.8%)	133 (10.8%)	152 (12.3%)*	165 (13.4%)*
BMI (kg/m^2^)	25.68±3.73	25.34±4.06	25.66±3.65	25.78±3.63	25.95±3.55
WC (cm)	99.15±7.58	97.85±7.88	98.82±7.28*	99.56±7.53*	100.40±7.39*
Waist-hip ratio	0.87±0.07	0.86±0.07	0.86±0.06	0.87±0.06	0.88±0.06*
Systolic BP (mm Hg)	128.15±18.58	123.52±17.79	125.45±17.98*	128.88±18.50*	132.75±19.08*
Diastolic BP (mm Hg)	77.87±10.57	77.85±10.58	77.47±10.38	78.35±10.37	77.82±10.93
Total cholesterol (mmol/L)	4.97±0.96	4.70±0.93	4.92±0.93*	5.08±0.92*	5.20±0.99**
Triglyceride (mmol/L)	1.38 (0.96–2.01)	1.23 (0.85–1.82)	1.32 (0.93–1.96)*	1.39 (1.00–2.04)*	1.51 (1.11–2.16)**
LDL cholesterol (mmol/L)	2.81±0.77	2.64±0.75	2.75±0.77*	2.85±0.74*	2.99±0.78**
HDL cholesterol (mmol/L)	1.42±0.35	1.43±0.34	1.42±0.55*	1.41±0.26*	1.38±0.36*
Fasting glucose (mmol/L)	5.27±1.36	5.30±1.61	5.22±1.32	5.26±1.17	5.29±1.32
Uric acid (mmol/L)	287.80±77.95	258.59±71.32	280.37±72.89**	291.86±73.75**	320.40±80.58**

Note: Characteristics are reported as percentages for categorical variables and means (±SD) or medians (with interquartile range) for continuous variables. The study participants were divided into four groups based on eGFR quartiles ((≥102.35, 92.42–102.34, 83.67–92.41, and ≤83.66 mL/min/ 1.73 m^2^). Categorical variables are presented as counts and percentages. The values outside the parentheses are the number of subjects, and the prevalence is presented in parentheses.

The first quartile of eGFR was used as the reference.

* p<0.05 vs. Quartile 1.

** p<0.01 vs. Quartile 1.

Abbreviations: eGFR, estimated glomerular filtration rate; BMI, body mass index; WC, waist circumference; BP, blood pressure; HDL, high-density lipoprotein; LDL, low-density lipoprotein.

### The association of HDL-C with eGFR in all participants

We performed univariate and multivariate analysis of the relationship between HDL-C and eGFR as a continuous variable in all participants. Table 2 shows the results of Pearson’s correlation and multivariable linear regression analyses. The results of Pearson’s correlation analysis indicated that HDL-C levels were associated with eGFR (r=0.16, P<0.001). In multivariate linear regression analysis, HDL-C levels were independently associated with eGFR. In addition, male gender, age, diabetes mellitus, BMI, WC, WHR, and triglyceride, LDL-C, and uric acid levels were independently associated with eGFR.

**Table 2:** Pearson’s correlation and multiple linear regression analyses of the association between HDL-C and eGFR.

	**Univariate**		**Multivariate**	
**Characteristics**	**r**	**P**	**β**	**P**
Male	0.002	0.830	−3.654	<0.001
Age	−0.418	<0.001	−0.453	<0.001
Current smoking	−0.014	0.173	0.204	0.673
hypertension	−0.139	<0.001	0.550	0.289
Diabetes mellitus	0.063	<0.001	3.229	<0.001
BMI (kg/m^2^)	−0.070	<0.001	0.302	0.001
WC (cm)	−0.157	<0.001	−0.127	0.008
Waist-hip ratio	−0.121	<0.001	11.566	0.035
Systolic BP (mm Hg)	−0.159	<0.001	0.004	0.786
Diastolic BP(mm Hg)	−0.011	0.293	0.034	0.186
Triglyceride (mmol/L)	−0.084	<.001	1.620	<0.001
LDL cholesterol (mmol/L)	−0.171	<.001	8.361	<0.001
HDL cholesterol (mmol/L)	0.162	**<0.001**	6.399	**<0.001**
Uric acid (mmol/L)	−0.303	<0.001	−0.061	<0.001
Fasting glucose (mmol/L)	−0.002	0.872	0.177	0.250

Abbreviations: BMI, body mass index; BP, blood pressure; HDL, high-density lipoprotein; LDL, low-density lipoprotein; eGFR, estimated glomerular filtration rate

The relationship between HDL-C and different quartile of eGFR among the participants is shown in Table 3. A stepwise logistic regression model was created, and quartile 1 of eGFR was used as the reference. The results illustrated that after adjustment for all confounders including age, gender, smoking status, a history of hypertension and diabetes mellitus, SBP, DBP, triglyceride, LDL-C, fasting glucose, and uric acid (model 4), HDL-C was independently associated with all quartiles of eGFR. Furthermore, the OR decreased gradually with lower eGFR values (quartiles 2–4).

**Table 3:** Association between HDL-C and eGFR in all participants.

	**Quartile 1**	**Quartile 2**	**Quartile 3**	**Quartile 4**
	**≥102.35**	**92.42–102.34**	**83.67–92.41**	**≤83.66**
**Model 1**				
Odds ratio	1	0.97	1.00	0.72
95% CI	Reference	0.74–1.27	0.75–1.33	0.51–1.02
P		0.836	0.979	0.063
**Model 2**				
Odds ratio	1	0.96	1.00	0.71
95% CI	Reference	0.73–1.26	0.75–1.34	0.50–1.01
P		0.770	0.999	0.058
**Model 3**				
Odds ratio	1	1.15	1.29	1.00
95% CI	Reference	0.85–1.55	0.94–1.78	0.68–1.46
P		0.370	0.116	0.982
**Model 4**				
Odds ratio	1	0.41	0.29	0.15
95% CI	Reference	0.25-0.66	0.17–0.48	0.08–0.28
P		**<0.001**	**<0.001**	**<0.001**

Note:

Model 1: Adjusted for age and gender.

Model 2: Adjusted for model 1 variables plus smoking status, history of hypertension, and history of diabetes mellitus.

Model 3: Adjusted for model 2 variables plus BMI, WC, WHR, SBP, and DBP

Model 4: Adjusted for with all the variables.

## Discussion

This is the first study to investigate the relationship between HDL-C and kidney function in a community-based population in China. In this study, we demonstrated that HDL-C levels were associated with eGFR in a community-based general population. Furthermore, after adjustment for all confounders (model 4), HDL-C was independently associated with all quartiles of eGFR among the participants, and the OR decreased gradually as the eGFR decreased (quartiles 2 to 4). However, HDL-C was not independently associated with eGFR when we adjusted for select confounders in a stepwise manner. Therefore, these results indicate that the independent relationship between low HDL-C levels and reduced kidney function is potentially influenced by confounders such as age, blood pressure, and lipid parameters. Moreover, this correlation should be enhanced gradually in all participants as eGFR declines. 

Some previous studies revealed a relationship between HDL-C and kidney function. However, these studies were overwhelmingly conducted in patients with ESRD or those receiving hemodialysis. Recently, a study reported that 176 patients with CKD (eGFR=50.3±29.1 mL/min/1.73 m^2^) were recruited and followed up for up to 84 months. The result of this cross-sectional study demonstrated that low HDL-C levels were associated with reduced eGFR. At follow-up, low HDL-C levels were associated with earlier entry in dialysis or doubling of the plasma creatinine level (P=0.017); HDL-C was the only lipid parameter that affected the progression of CKD [hazard ratio (HR)=0.951; 95% CI, 0.917–0.986; P=0.007] independently of the presence of diabetes [17]. However, epidemiological data concerning the relationship between HDL-C and kidney function in the general population are rare. In the present study, we investigated the correlation between HDL-C and eGFR in a community-based population with roughly normal kidney function (the number of the subjects with eGFR <60 mL/min/1.73 m^2^ was 93). The results revealed that low HDL-C levels were independently associated with reduced eGFR in the general population. 

Although, our study also confirmed the correlation between eGFR and HDL-C in the general population; this correlation differs from that between eGFR and HDL-C in patients with moderate to severe kidney dysfunction. In patients with ESRD, reduced kidney function results in the dysregulation of HDL-C metabolism [18], and reduced HDL-C levels are a hallmark of ESRD-related dyslipidemia. In this type of correlation, reduced kidney function could be the cause of decreased HDL-C levels. By contrast, in the general population with roughly normal kidney function, the cause of the association of HDL-C with eGFR may be the reduced hepatoprotective effects of lower HDL-C levels. Several potential mechanisms may explain the link between low HDL-C levels and reduced eGFR. First, low HDL-C levels represent an established risk factor for atherosclerosis. This is a primary cause of reduced kidney function due to renal artery stenosis [19]. Second, as our previous study confirmed, low HDL-C levels are independent associated with arterial stiffness [20], which is a major cause of reduced kidney function [21]. Third, low HDL-C levels may influence microvascular disease affecting the kidneys. Morton et al. [22] reported the association of vascular risk factors with complications of type 2 diabetes. After a median of 5 years of follow-up, compared with patients in the highest tertile, those in the lowest tertile had a 17% higher risk of microvascular disease (adjusted HR=1.17; 95% CI, 1.06–1.28; P=0.001) after adjustment for potential confounders. The HDL-C level is an independent risk factor for the development of microvascular disease affecting the kidney. Finally, HDL has potential pleiotropic effects such as antioxidative, anti-inflammatory, and antiapoptotic effects, and the pleiotropic effects of HDL possibly protect kidney function [23]. 

To better understand the correlation between HDL-C and eGFR, eGFR values were classified by quartiles, and correlative confounders were adjusted stepwise in the present study. The results indicated that HDL-C was independently associated with all quartiles of eGFR after adjustment for all confounders (model 4), and this independent association was not apparent in the other models (model 1, 2, 3). This result implied that other confounders such as age, blood pressure, and lipid parameters may affect kidney function in the general population with roughly normal eGFR values. 

There are several limitations in the present study. First, because of the cross-sectional design and its inherent limitations, the present study cannot determine causal relationships between the associations. Accordingly, our observations need confirmation in longitudinal and interventional studies. Second, we use creatinine-based eGFR as an index of kidney function. Creatinine is a byproduct of muscle mass, and it is less accurate for assessing kidney function in older adults, who often have reduced muscle mass [24]. There were approximately 1500 subjects who were older than 60 years in the present study, which may have affected the results. Third, the study population is Chinese, and kidney function was estimated according to CMDRD. Although the present study provides important ethnic data to clarify the relationship between HDL-C and eGFR, extrapolation of our results to other demographic groups should be done with caution. Fourth, although the results were adjusted for multiple covariates that may be associated with eGFR values, the possibility of residual confounding remains. 

## Conclusion

In conclusion, HDL-C is independently associated with eGFR after adjustment for multiple covariates in the general population. Moreover, this correlation should be enhanced gradually as eGFR declines. Further, large and well-conducted studies are urgently required to provide more definitive evidence of this correlation.
